# Dynamic Cerebral Autoregulation Remains Stable During the Daytime (8 a.m. to 8 p.m.) in Healthy Adults

**DOI:** 10.3389/fphys.2018.01642

**Published:** 2018-11-20

**Authors:** Wei-tong Guo, Hongyin Ma, Jia Liu, Zhen-Ni Guo, Yi Yang

**Affiliations:** ^1^Department of Neurology, The First Hospital of Jilin University, Changchun, China; ^2^Institute of Advanced Computing and Digital Engineering, Shenzhen Institutes of Advanced Technology, Chinese Academy of Sciences, University Town of Shenzhen, Shenzhen, China; ^3^Department of Neurology, Clinical Trial and Research Center for Stroke, The First Hospital of Jilin University, Changchun, China

**Keywords:** dynamic cerebral autoregulation, blood pressure, cerebral blood flow, daily rhythm, cerebrovascular disease

## Abstract

Many functions of the human body possess a daily rhythm, disruptions of which often lead to disease. Dynamic cerebral autoregulation (dCA) stabilizes the cerebral blood flow to prompt normal neural function. However, whether dCA is stable across the day remains unknown. This study aimed to investigate the daily rhythm of dCA. Fifty-one healthy adults (38.294 ± 13.279 years, 40 females) were recruited and received six dCA measurements per individual that were conducted at predefined time points: 8:00, 9:00, 11:00, 14:00, 17:00, and 20:00. Although the blood pressure fluctuated significantly, there was no statistical difference in phase difference and gain (autoregulatory parameters) across the six time points. This study demonstrates that dCA remains stable during the interval from 8 a.m. to 8 p.m. and underscores the importance of stable dCA in maintaining cerebral blood flow and neural function.

## Introduction

Many functions of the human body have their own circadian rhythms and change over the course of a day. Dynamic cerebral autoregulation (dCA) is a process to maintain cerebral blood perfusion at an appropriate state via regulating cerebral vasculature (Budohoski et al., 2013). Cerebral autoregulation typically works with a mean arterial pressure (MAP) in the range of 60 and 150 mm Hg in normal conditions (Paulson et al., 1990). Previous studies have shown that patients with hemorrhagic stroke (Ma et al., 2016), ischemic stroke (Guo et al., 2014; Salinet et al., 2018), generalized anxiety (Guo et al., 2018), epilepsy (Lv et al., 2018), and Alzheimer's disease (Shekhar et al., 2017) have impaired dCA. Cerebral autoregulation can be affected many factors, such as CO_2_ (Meng and Gelb, 2015), nitric oxide (Guo et al., 2016) and altitude level (Jansen et al., 2007). However, the daily rhythm of dCA and whether the time of measurement interferes with the evaluation of dCA are unclear. This study aimed to determine the rhythm of dCA during the interval from 8 a.m. to 8 p.m. in healthy adults.

## Material and methods

### Study protocol

The study performed six dCA measurements at six predefined time points: 8:00, 9:00, 11:00, 14:00, 17:00, and 20:00. All the subjects had no history of chronic diseases or acute infections within the 2 weeks before beginning the study. Subjects with intracranial and/or extracranial major vascular stenosis/occlusion diagnosed by a transcranial Doppler (EMS-9PB, Delica, China) and carotid ultrasound (IU22, Phillips, Andover, MA) were excluded. All the subjects were informed of the study protocol 1 day in advance. Subjects were instructed to refrain from caffeinated drinks and alcohol ingestion for 24 h before the examination (Claassen et al., 2016). All the subjects were non-smokers. The subjects were asked to avoid strenuous exercise, caffeine, and alcohol during the whole research process. The study was approved by the Ethics Committee of the First Hospital of Jilin University. Written consent was provided by all participants.

### DCA measurement

Measurements were performed in the specific examination room by a professional technician. The room was quiet and had a controlled temperature of 20°C to 24°C. The subjects were asked to take a relaxed supine position for 10 min. First, the technician measured the arterial blood pressure (ABP) at the brachial artery by an automatic blood pressure monitor (Omron 711, Japan). The continuous ABP was measured non-invasively using a servo-controlled plethysmograph (Finometer Model 1, FMS, Netherlands) at the middle finger. Simultaneously, two 2 mHz transcranial Doppler probes were placed over the temporal windows to monitor in real-time the bilateral middle cerebral arteries at a depth of 45–60 mm. the probes were fixed with a customized head frame to make sure cerebral blood flow velocity (CBFV) was continuously and stably measured. CBFV and continuous ABP were recorded simultaneously from each subject for 10 min. All data were recorded for further assessment and analysis.

### Data analysis

Data of ABP and CBFV were acquired using MATLAB (MathWorks, Inc., US). The dynamic relationship between ABP and CBFV was analyzed by transfer function analysis (TFA) as follows (Claassen et al., 2016). A cross-correlation function between ABP and CBFV was used to align the data on in order to eliminate the possible time lags. An anti-alias filter, a third-order Butterworth low-pass filter, with cutoff frequency at 0.5 Hz was applied so as to down-sample the data to 1 Hz. Welch's method was employed to estimate the autospectrum of ABP, *S*_*xx*_(*f*), and the cross-spectrum of ABP and CBFV, *S*_*xy*_(*f*), in frequency domain by averaging the periodograms of the down-sampled ABP and CBFV with a 50% overlapped hamming window of 90 s. The transfer function, *H*(*f*), was then deviated as:

(1) $$\begin{array}{r} H\left(f\right)=\frac{S_{xy}\left(f\right)}{\;S_{xx}\left(f\right).} \end{array}$$

Gain and phase difference (PD) can then be calculated from (1) by Equation (2) and (3), respectively, as:

(2) $$\begin{array}{rcl} \left|H\left(f\right)\right| & = & \sqrt{\left\{|H_{R}\left(f\right){|}^{2}+|H_{I}\left(f\right){|}^{2}\right\}}, \end{array}$$

(3) $$\begin{array}{rcl} \theta & = & tan^{-1}\left[H_{I}\left(f\right)/H_{R}\left(f\right)\right]. \end{array}$$

where R and I denote the real and imaginary parts of the transfer function, respectively. Phase difference (PD), gain, and coherence function within a 0.06–0.12 Hz frequency range were then derived from TFA to evaluate dCA. A low value of PD indicates that CBFV follows the changes of ABP passively, whereas a high value of PD suggests that CBFV is actively regulated against the fluctuations of ABP (van Beek et al., 2008). Due to the fact that TFA is a linear model-based method, signals with low coherence between ABP and CBFV (≤0.40) were excluded from further statistical analysis.

### Statistical analysis

The statistical analysis of the data was conducted using IBM SPSS Statistics 24.0 (Armonk, NY, United States), and a two-tailed *P*-value < 0.05 was considered to be statistically significant. The distribution of data was assessed using a one-sample Kolmogorov—Smirnov test. Data are shown as mean ± standard deviation (SD) for normally distributed continuous variables. Repeated measurement analysis of variance was performed for comparing differences in observed values at different time points.

## Results

**Figure 1:**
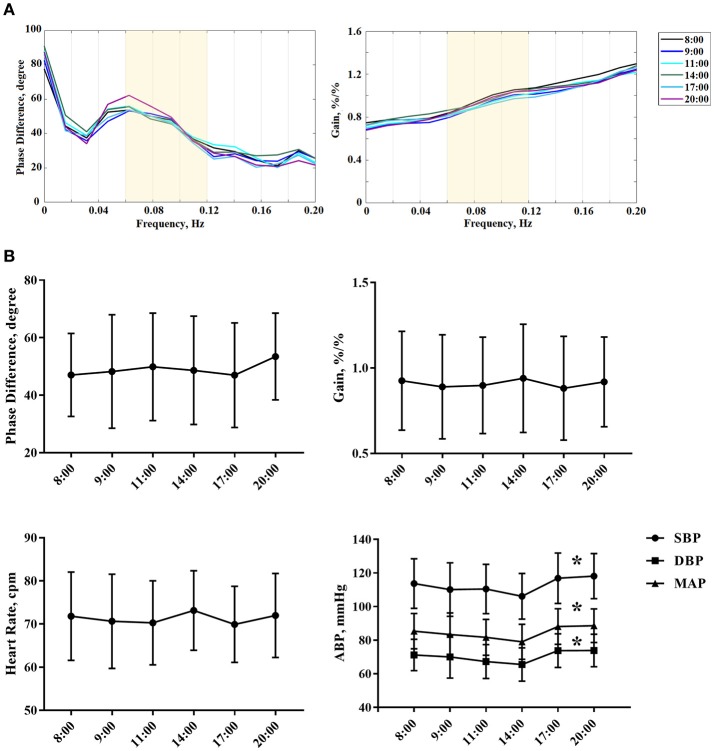
**(A)** The autoregulatory parameter (phase difference and gain) derived from the transfer function analysis and estimated over a range (0.06–0.12 Hz, the yellow area) at 8:00, 9:00, 11:00, 14:00 ,17:00, and 20:00. Each colored line denotes the averaged PD or gain estimated from all subjects at one particular time point that we collected the data. **(B)** Statistical analysis of serial dCA, arterial blood pressure, and heart rate of serial measurements. The solid point represents means, and the whiskers denote standard deviation. ^*^P < 0.05 for comparison of different time points by repeated measurement analysis of variance. HR, indicates heart rate; cpm, counts per minutes; SBP, systolic blood pressure; DBP, diastolic blood pressure; MAP, mean arterial pressure.

The current study enrolled 51 healthy adults (38.294 ± 13.279 years, 40 females). DCA was measured in each subject for six times according to the preset procedure. Thus, the study included 306 records in total for dCA analysis. The coherence of all records was over 0.40. ABP and heart rate of serial measurements are presented in Table 1. ABP including systolic blood pressure (SBP), diastolic blood pressure (DBP), and mean arterial pressure (MAP) was statistically significant at different time points (*P* < 0.001, Figure 1, Table 1). Heart rate remained stable during the whole 12 h in a day (Figure 1, Table 1). The results of female or male independent analysis and whole subject analysis were consistent (Table 1).

**Table 1:** Dynamic cerebral autoregulation parameter (phase difference, gain), mean arterial blood pressure, and heart rate and statistical results.

		**Measurements at 8:00**	**Measurements at 9:00**	**Measurements at 11:00**	**Measurements at 14:00**	**Measurements at 17:00**	**Measurements at 20:00**	**F**	**p**
Both sexes	Phase difference, degree	48.169 ± 15.488	48.242 ± 19.733	49.164 ± 17.741	49.213 ± 17.901	46.069 ± 17.846	52.427 ± 15.648	1.379	0.233
	Gain, %/%	0.925 ± 0.289	0.890 ± 0.304	0.898 ± 0.282	0.940 ± 0.317	0.881 ± 0.304	0.919 ± 0.262	0.783	0.563
	SBP, mmHg	113.7 ± 14.7	110.1 ± 15.9	110.5 ± 14.7	106.1 ± 13.6	116.8 ± 15.0	118.1 ± 13.4	15.405	<0.001
	DBP, mmHg	71.2 ± 9.4	70.0 ± 12.6	67.3 ± 10.1	65.5 ± 10.0	73.8 ± 10.1	73.9 ± 9.7	14.695	<0.001
	MAP, mmHg	85.4 ± 10.5	83.4 ± 12.9	81.7 ± 10.7	79.0 ± 10.4	88.1 ± 10.6	88.6 ± 10.0	19.663	<0.001
	HR, cpm	71.8 ± 10.2	70.6 ± 10.9	70.3 ± 9.7	73.1 ± 9.2	69.9 ± 8.8	72.0 ± 9.7	2.374	0.052
Male	Phase difference, degree	46.372 ± 18.645	37.907 ± 16.466	41.044 ± 23.781	43.640 ± 16.175	40.175 ± 18.317	50.130 ± 19.258	2.378	0.052
	Gain, %/%	0.918 ± 0.194	0.899 ± 0.252	0.849 ± 0.209	0.912 ± 0.241	0.823 ± 0.224	0.874 ± 0.205	0.840	0.528
	SBP, mmHg	121.7 ± 9.1	120.5 ± 9.6	117.9 ± 12.1	118.0 ± 7.8	126.6 ± 11.8	128.1 ± 6.6	4.992	0.008
	DBP, mmHg	74.0 ± 5.7	76.1 ± 10.8	70.5 ± 6.5	71.3 ± 9.3	79.2 ± 11.1	77.2 ± 7.0	2.944	0.021
	MAP, mmHg	89.9 ± 5.9	90.9 ± 10.1	86.3 ± 7.8	86.8 ± 8.4	95.0 ± 10.8	94.2 ± 6.1	4.438	0.002
	HR, cpm	72.2 ± 13.4	72.7 ± 9.0	67.9 ± 7.5	74.9 ± 7.3	69.4 ± 6.2	69.9 ± 9.5	1.675	0.158
Female	Phase difference, degree	48.663 ± 14.738	51.084 ± 19.782	51.397 ± 15.324	50.745 ± 18.236	47.689 ± 17.601	53.059 ± 14.720	0858	0.510
	Gain, %/%	0.928 ± 0.313	0.887 ± 0.320	0.912 ± 0.300	0.947 ± 0.337	0.897 ± 0.323	0.931 ± 0.277	0.537	0.748
	SBP, mmHg	111.5 ± 15.3	107.3 ± 16.2	108.5 ± 14.8	102.9 ± 13.0	114.2 ± 14.8	115.4 ± 13.6	11.620	<0.001
	DBP, mmHg	70.4 ± 10.1	68.4 ± 12.6	66.4 ± 10.8	63.9 ± 9.7	72.3 ± 9.4	73.0 ± 10.2	12.449	<0.001
	MAP, mmHg	84.1 ± 11.2	81.3 ± 12.9	80.4 ± 11.2	76.9 ± 10.0	86.2 ± 9.9	87.1 ± 10.4	15.994	<0.001
	HR, cpm	71.7 ± 9.4	70.1 ± 11.4	70.9 ± 10.2	72.7 ± 9.7	70.1 ± 9.5	72.6 ± 9.8	1.854	0.104

*SBP, systolic blood pressure; DBP, diastolic blood pressure; MAP, mean arterial pressure; HR, heart rate; cpm, counts per minutes*.

### DCA

From 8:00 to 20:00, the PD was not significantly changed within 12 h (*P* = 0.233). Furthermore, the PD and gain showed no difference between left and right hemispheres at all the time points (*P* = 0.573, 0.388, 0.854, 0.263, 0.200, 0.665). Interestingly, the PD tended to be higher at 20:00 compared with the value at 8:00, but it was not statistically significant (Table 1, Figure 1). The gain did not significantly differ among all study time points (Table 1, Figure 1).

## Discussion

The present study investigated the daily rhythm of dCA and the relationship between ABP and dCA. The major finding of the study was that dCA remains at a stable level during the daytime. Furthermore, dCA did not fluctuate following the changes of ABP.

Clinically, dCA measurement can correctly reflect most aspects of the autoregulatory response (Tiecks et al., 1995). Previous studies have shown that dCA was affected by the sympathetic nerve (Hamner et al., 2010), cholinergic nerve (Hamner et al., 2012), myogenic mechanisms (Tan et al., 2013), and metabolic control (Payne, 2018). Although these mechanisms have their own change rules, we showed that dCA is maintained at a stable level. The present study may indicate that the daily rhythm of dCA is independent of the change rule of one single mechanism.

Cerebral autoregulation functions to stabilize cerebral blood flow and metabolism while the ABP changes (Lassen, 1959). When dCA was impaired, the cerebral blood flow would change with fluctuations in blood pressure, leading to cerebrovascular disease. ABP fluctuation has been found to be a potent risk factor for cerebrovascular disease (Yano and Kario, 2012). Due to the morning blood pressure surge, most strokes occur in the morning hours (Marler et al., 1989; Yano and Kario, 2012). Similar to previous studies (Atkinson et al., 2010; Douma and Gumz, 2018), the present study reproduced the finding of ABP fluctuations. However, we found that dCA was independent of ABP fluctuations during the daytime.

Our analysis indicated that healthy adults have stable and functional dCA to maintain brain perfusion, similar to the findings of a previous study (Brys et al., 2003). Chi et al have demonstrated that dCA was interchangeable and effective by assessing from the internal carotid artery compared with that from the middle cerebral artery (Chi et al., 2017). Also, dCA assessed by using a 5 min recording was identical with that using a 10 min recording in the clinical application (Chi et al., 2018). Despite the stability of different recording time and different measuring locations, we have discovered the stable rhythm of dCA during the 8:00–20:00 interval. And, conversely to the abundant literature showing differences in cerebral blood flow between sexes (Barnes, 2017) and the influence of sex on circadian rhythms (Yan and Silver, 2016), the present study has shown the same results for both sexes.

In the literature, dCA was reported to be reduced at 6 a.m.−8 a.m. (Ainslie et al., 2007), which is different from the present study. We speculate that the differences might arise from three reasons: (1) different detection time of cerebral autoregulation: Ainslie et al performed the morning dCA measurement in the early morning (6 a.m.−8 a.m.), while our study performed the morning dCA measurement at 8 AM. Because we don't know the differences between “early morning” and “morning” of the dCA, we cannot exclude the difference caused by the detection time points; (2) the sex of the subjects: Ainslie et al recruited all male subjects, while the present study recruited both male and female subjects; (3) the age of the subjects; Ainslie et al recruited young subjects with a mean age of 25. However, our study recruited middle-aged subjects with a mean age of 38.

The present study shows that dCA is a reliable indicator and remains at a stable level during the daytime and was not affected by fluctuations of ABP. Thus, given its stability and reliability, it possible to assist in diagnosing and assessing the cerebrovascular function of cerebrovascular diseases, generalized anxiety, epilepsy and Alzheimer's disease. Simultaneously, the dCA also can be used to evaluate the treatment effect of the above disease.

However, there are several limitations to the study. The current study lacks the nocturnal and early morning rhythm of dCA. The main reason is that monitoring dCA requires the subjects to stay awake. If night monitoring is carried out, subjects may not sleep well, which may affect daytime results. Furthermore, due to the nature of the observational study, we did not collect the blood samples at different time points to test the changes of biomarkers. These questions should be pursued in future studies.

In summary, the rhythm of dCA keep was steady during the 8 a.m.−8 a.m. interval in healthy adults, and it is not influenced by the fluctuations of ABP. For evaluating dCA, one random measurement of dCA is reliable.

## Author contributions

WG design the study, acquired the data, analyzed and interpreted of data, drafted the manuscript. Z-NG analyzed and interpreted of data and revised the manuscript. JL analyzed and interpreted of data and obtained funding. HM acquired the data and revised the manuscript. YY studied concept and designed the study, critical revised the manuscript, supervised the study and obtained funding.

### Conflict of interest statement

The authors declare that the research was conducted in the absence of any commercial or financial relationships that could be construed as a potential conflict of interest.
